# Repeated Yueju, But Not Fluoxetine, Induced Sustained Antidepressant Activity in a Mouse Model of Chronic Learned Helplessness: Involvement of CaMKII Signaling in the Hippocampus

**DOI:** 10.1155/2022/1442578

**Published:** 2022-02-24

**Authors:** Zhilu Zou, Jiaru Huang, Qingqing Yang, Yuxuan Zhang, Bo Xu, Ping Wang, Gang Chen

**Affiliations:** ^1^School of Basic Medicine, Hubei University of Chinese Medicine, Wuhan 430065, China; ^2^Interdisciplinary Institute for Personalized Medicine in Brain Disorders, Jinan University, Guangzhou 510632, China; ^3^Departments of Psychiatry & Clinical and Translational Institute of Psychiatric Disorders, First Affiliated Hospital of Jinan University, Guangzhou 510632, China; ^4^Co-innovation Center of Neurodegeneration, Nantong University, Nantong 226001, China; ^5^Center for Translational Systems Biology and Neuroscience, Key Laboratory of Integrative Biomedicine for Brain Diseases, Nanjing University of Chinese Medicine, Nanjing 210023, China

## Abstract

**Objective:**

Depression is characterized with long disease length, whereas one major disadvantage of current mainstream treatment of depression is a high rate of relapse and recurrence. A sustained antidepressant activity is proposed to facilitate the prevention of relapse/recurrence. Here we compared the long-term antidepressant effect of Yueju, a traditional Chinese medicine formula, and a conventional antidepressant, fluoxetine, as well as revealing the underlying mechanism of long-term antidepressant effect of Yueju.

**Methods:**

Clinical long-term depression condition was modelled by using chronic learned helplessness (cLH) protocol in ICR strain mice. The short-term and long-term antidepressant effects of drugs were assessed with learned helplessness (LH), tail suspension test (TST), forced swim test (FST), and novelty-suppressed feeding (NSF) test. The expression of PKA, CaMKII signaling, and NR1, the NMDA receptor subunit, in hippocampus was determined. A CaMKII inhibitor (KN-62) was used to assess the role of CaMKII signaling in antidepressant effects of Yueju or fluoxetine.

**Results:**

In the mice exposed to chronic learned helplessness (cLH) procedure, administration of Yueju or fluoxetine for 3 weeks elicited comparable antidepressant effects, indicated by learned helplessness test, as well as TST and NSF. However, 5 days after termination of the 3-week-long drug administration, only mice previously treated with Yueju still showed the alleviation of depressive-like behaviors. At this time, the downregulation of PKA and p-CaMKII/CaMKII and upregulation of NMDA receptor subunit NR1 in the hippocampus were normalized in animals previously treated with Yueju. In contrast, none of the expressions of these proteins were changed in mice previously treated with fluoxetine. Interestingly, an administration of KN-62 blunted the antidepressant effect of Yueju.

**Conclusion:**

These findings showed the sustained antidepressant efficacy of chronic treatment with routine dose of Yueju and the CaMKII signaling activation may play a critical role in the sustained antidepressant response.

## 1. Introduction

Depression is a common psychiatric disorder characterized by persistent and significant low mood and a high suicidal tendency in severe cases [1]. It is a disease with high incidence, high disability rate, and high recurrence rate [2]. The episode of depression lasts for at least 2 weeks, and a substantial proportion of its burden arises through relapses and chronic courses: more than half of those with a first episode of depression go on to have a second one, and the majority of them have more episodes [3, 4]. Unfortunately, current mainstream antidepressants have a long treatment time and a considerably high recurrence rate even after receiving adequate doses and duration of treatment [5]. The recurrence risk has been shown to increase with 16% after each successive episode [6, 7]. Development of antidepressant drugs capable of reducing the relapse/recurrence is critical for improvement of the clinical treatment of depression.

Currently, selective serotonin reuptake inhibitors (SSRIs) and selective norepinephrine inhibitors (SNRIs) are most frequently used in the treatment of depression [8, 9]. Besides high recurrence rates, they also have limitations in substantial late onset for a therapeutic response (weeks to months) and side effects at beginning and/or after a long-term use (such as decreased libido and weight increases) [7, 10, 11]. Recent studies indicate that ketamine, an anesthetic medication, confers fast-onset therapeutic effects within hours, which may last for several days [12, 13]. However, treatment with a single dose of ketamine is also very prone to relapse. Moreover, ketamine has toxic and abuse potential [14, 15]. Great efforts thus have been made to use ketamine as the prototype to develop novel antidepressant “Yueju,” a traditional herbal medicine formulated 800 years ago, which is now frequently prescribed to treat depression, anxiety, and irritability [16]. Recently, it has been demonstrated that Yueju with a high dose displayed rapid antidepressant effects in both preclinical and clinical studies [17, 18], whereas a conventional low dose of Yueju also elicited an antidepressant effect. Interestingly, in a chronic learned helplessness model, two-week treatment with a low dose of Yueju showed superior antidepressant activity than fluoxetine, a classic SSRI [19]. In this study, we identified the major compounds of Yueju pills by using HPLC, in which 11 compounds were identified, including chlorogenic acid, geniposide, ferulic acid, senkyunolide I, senkyunolide A, n-butylphthalide, ligustilide, 3-butylidenephthalide, and atractylodin (shown in Supplementary Figure S1), and the structures of major compounds are shown in Supplementary Figure S2. Geniposide, one of the major compounds in Yueju pill extracts, showed the antidepressant activity in a previous study [20, 21].

Several molecular signaling features were revealed to be shared by Yueju and ketamine, including instant increase in BDNF in the hippocampus and inhibition of N-methyl-D-aspartate (NMDA) receptors (NMDAR) in the hippocampus and prefrontal cortex. On the other hand, rapid antidepressant activity of Yueju, but not ketamine, was dependent on protein kinase A- (PKA-) CREB signaling [22]. PKA-CREB signaling regulates the expression of genes that promote synaptic and neural plasticity, such as PSD95 [23]. Chronic treatment with fluoxetine enhances cAMP levels and then activates a PKA-CREB signal pathway in the hippocampus of mice [24, 25]. Two-week treatment with low dose of Yueju improved the deficiency in PKA/CREB/BDNF and decreased expressions of NMDA receptor subunits in the hippocampus in a chronic learned helplessness paradigm, whereas repeated fluoxetine only reversed some but not all of these signaling deficits, in parallel with the more depressive symptoms alleviated by Yueju than by fluoxetine [19]. Thus, more comprehensive and persistent improvement in the molecular deficits may account for the better antidepressant effects.

We previously established a long-term learned helplessness depression model, which reliably reproduced the chronic feature of depression [19]. Here, we used this model to test the sustained antidepressant effects after termination of chronic treatment with Yueju or fluoxetine. This sustained antidepressant effect may indicate the potential to reduce relapse/recurrence of depression in the long-term depression model. We also assessed the signaling associated with the behavioral differences. We found, in addition to PKA-CREB and NMDA signaling, that the restoration of CaMKII signaling was closely associated with the sustained antidepressant effects. CaMKII dysfunction is involved in myriad neuropsychiatric disorders including depression [26–28]. Transient manipulation of CaMKII results in long-lasting effects on disease-related behaviors, implying that CaMKII may result in structural or transcriptional alterations that contribute to disease progression and duration [29, 30]. Therefore, we also examined the role of CaMKII activation in the antidepressant activity of Yueju or fluoxetine, using pharmacological intervention approach.

## 2. Materials and Methods

### 2.1. Animals

Male and female ICR mice were obtained from the Shanghai Sipur-Bikai Experimental Animal Co., Ltd. Mice aged approximately 6 weeks (18–24 g) were habituated to animal facilities for 7 days prior to behavioral testing. The animals were kept at room temperature of 25 ± 2°C, with 12 h light and dark cycle. Excluding the experimental time, mice were fed freely with food and water. All animal experiments were in accordance with the Guide for the Care and Use of Laboratory Animals approved by the Institutional Animal Care and Use Committee at Jinan University.

### 2.2. Drugs

The Yueju pills are supported by Huqingyu Tang Pharmaceutical Co., Ltd (Hangzhou, China). The pills were ground into powder and dissolved with 0.9% saline. Yueju is composed of the following: *Cyperus rotundus* L. (Xiang Fu), *Ligusticum chuanxiong* Hort. (Chuan Xiong), *Gardenia jasminoides* Ellis. (Zhi Zi), *Atractylodes lancea* (Thunb.) DC. (Cang Zhu), and Massa Fermentata (Shen Qu). The concentration of the solution was 0.125 g/mL. The drug dose was 1.25 g/kg/day. Fluoxetine (Sigma-Aldrich, St. Louis, MO, USA) was dissolved in 0.9% saline. The concentration of the solution was 0.0018 g/mL. The dose of fluoxetine was 18 mg/kg/day, according to previous study [30, 31]. KN-62 (1 *μ*g/site, a CaMKII inhibitor) was dissolved in saline with 1% DMSO. KN-62 was administered to mice by the intracerebroventricular (i.c.v.) route (5 *μ*L/site).

### 2.3. Behavioral Tests

All behavioral tests were performed between 9 : 00 and 17 : 00. Animals were transferred to the testing room and habituated to the room conditions for at least 1 hour before the beginning of the behavioral experiments. Mice were randomly assigned to groups, and behavioral testers were blinded to the experimental groups.

#### 2.3.1. Open Field Test

Mice were placed individually in open field arena (40 × 40 × 40 cm) and allowed to freely explore. A camera was mounted above to record locomotor activity. The entire test site was adjusted to uniform lighting. Mice were placed in the center of the arena and allowed to freely explore for 5 minutes. The total distance traveled and time spent in central area were measured. The test equipment was thoroughly wiped with 70% ethanol before being put into each experimental mouse.

### 2.4. Tail Suspension Test

The instrument consists of six chambers. The front of the box was open, with an attached vertical bar hanging down in the center. The mice were tied 1 cm from the tip of the tail to the vertical bar with tape, and the mice were suspended. A camera was placed in front of the TST box to record the behavior of the mice for 6 min. The immobility of the mice was measured during the last 4 min of the experiment.

### 2.5. Forced Swim Test

The mice were gently placed in a transparent Plexiglas tank with a height of 30 cm and a diameter of 10 cm. The glass tank was filled with water with a depth of 15 cm. The temperature of the water was 23–26°C. A video camera recorded the six min of swimming. The immobility of the mice was measured during the last 4 min of the experiment. In this test, the immobility time is defined as they passively float in the water.

#### 2.5.1. Novelty-Suppressed Feeding Test

After fasting for 18 h, the mice adapt to the experimental environment for 1 h and they were put in a new cage. Each mouse was put in the front of the cage, and the weighed grain was placed in the center of the cage. Each mouse was allowed to explore up to 10 min. The amount of food consumed in the home cage was taken as the weight of chow consumed in 10 min. Food consumption is the weight of chow consumed divided by the weight of the mice. The time mouse started to consume the food pellet was considered as the latency.

#### 2.5.2. Chronic Learned Helplessness (cLH) Protocol

Chronic learned helplessness (cLH) consists of two stages: training stage and testing stage. In the training stage, cLH paradigm was carried out as described previously [19] in a shuttle cage (40 × 10 × 13 cm) that was divided equally into two chambers. Mice were trained for LH induction by administering 120 scrambled, inescapable foot shocks (0.45 mA shock amplitude, 15 s duration, 18–44 s average interval) over a 1 h session. For the cLH protocol, mice were trained for 3 consecutive days, followed by two additional intermittent training days: day 8 and day 13. Control animals were exposed to the apparatus for the same period without receiving foot shocks.

In the testing stage, each mouse was given 30 shuttle escape trials with 3 s duration and 18–44 s intervals. The door was raised at the beginning of the shock, and each trial was terminated when the mouse moved into the nonshock compartment. Latency to escape and the number of escape failures were recorded automatically by software.

### 2.6. Stereotaxic Surgery and Microinjection

Mice were anesthetized and implanted with a guide cannula (3.3 mm) into lateral ventricle according to the procedure described previously with some modifications [19, 32, 33]. Mice were firstly anesthetized by using gaseous anesthesia with isoflurane using a mice anesthetics machine. The mice were fixed on the stereotaxic apparatus and kept in a neutral position, and the scalp was incised along the midline to expose the mouse skull. The skull surface was coated with 3% hydrogen peroxide. After the guide cannula was inserted into lateral ventricle (coordinates: 0.6 mm posterior and 1.1 mm lateral to the bregma), the dental cement was used to fill the area around the cannula and a dummy cannula was inserted into the guide cannula to maintain the cannula patency. Animals were individually housed, handled daily, and allowed to recover for 7 days after surgery.

All the mice that had been microinjected were awake and free-moving. On the experimental day, a PE tubing connected to a 5 *μ*L syringe was inserted into the guide cannula and extended 1 mm beyond the tip. Drugs or vehicle was infused in a volume of 5 *μ*L over 5 minutes. An additional 5 min was allowed for the diffusion and prevention of backflow through the needle track before the injector was withdrawn. 30 min after Yueju or fluoxetine administration, mice were subjected to the OFT, TST, FST, and NSF.

### 2.7. Western Blot

Protein was extracted from mice's hippocampal tissue. Protein lysates were separated by 12% SDS-PAGE electrophoresis and transferred onto polyvinylidene difluoride (PVDF) membranes. After blocking with 3% BSA for 1 h, the membranes were incubated with primary antibodies. CAMKII (Cell Signaling Technology, 7546, 1 : 500), P-CaMK-II (Cell Signaling Technology, 9546, 1 : 500), P-CREB (Cell Signaling Technology, 9198 s, 1 : 500), CREB (Cell Signaling Technology, 9197, 1 : 500), PKA (Proteintech, 55388-1-AP, 1 : 1000), NMDAR1 (Cell Signaling Technology, 5104 s, 1 : 1000), BDNF (Cell Signaling Technology, 47808, 1 : 1000), PSD95 (Cell Signaling Technology, #2507, 1 : 1000), GAPDH (Proteintech, 6004-1-lg, 1 : 2000) and Tubulin (Proteintech, 10094-1-AP, 1 : 2000) were used at 4°C overnight. The next day, blots were washed 4 times in TBST, followed by incubation with secondary antibodies for 1 h. After washing for 3 times, the blots were visualized using the Super Signal West Pico Chemiluminescent Substrate (Thermo Fisher Scientific Inc.). BDNF and pro-BDNF were normalized to tubulin bands, and P-CERB and total CREB bands were taken as a ratio of tubulin bands. All experiments were performed 3 times.

### 2.8. The HPLC Fingerprint of Yueju Pill Extracts

The sample was prepared as the following procedure: 2 g of Yueju pills was dissolved in 10 mL of 70% methanol and extracted for 30 min. The sample was analyzed by using high performance liquid chromatography (HPLC). The HPLC analysis was performed on a Waters 2695 system (Waters Corporation, Milford, MA, USA), consisting of a binary solvent delivery manager, an autosampler, and a PDA detector. Chromatographic separations were performed on Alltima C_18_ column (5 *μ*m, 250 × 4.6 mm). Flow rate and column temperature were set at 1 mL·min-1 and 30°C, respectively. A mobile phase system consisting of 0.1% formic acid in H_2_O (A)-Acetonitrile (B) was applied with the following gradient program: 0–8 min, 90%A; 8–15 min, 90–80% A; 15–25 min, 80% A; 25–30 min, 80%–70% A; 30–38 min, 70% A; 38–43 min, 70%–50% A; 43–48 min, 50% A; 48–56 min, 50%–30% A; 56–59 min, 30% A; 59–65 min, 30%–0% A; 65–75 min, 0% A; and 75–80 min, 0%–90% A.

### 2.9. Statistics Analyses

Two-sample comparisons were carried out using the two-tailed Student's *t*-test; multiple comparisons were made using one-way ANOVA, followed by the Turkey's multiple comparison tests. Two-way ANOVA was used for the analysis of behavioral effects of KN-62 and Yueju or fluoxetine in the TST, FST, and OFT. All data are presented as mean ± SEM, and statistical significance was accepted at the 5% level unless otherwise indicated.

## 3. Results

### 3.1. Screening of Effective Antidepressant Dose of Yueju in ICR Strain Mice

**Figure 1:**
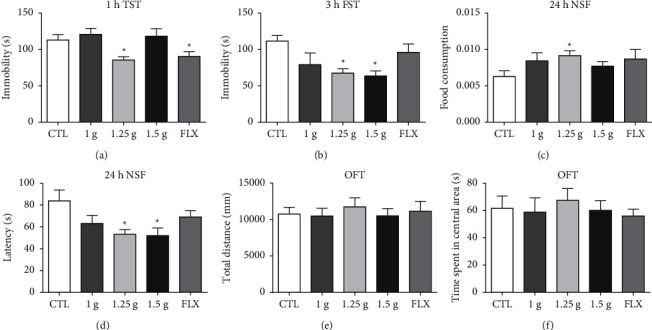
Effective dose of Yueju in inducing antidepressant effects in ICR mice. The doses of Yueju of 1 g/kg, 1.25 g/kg, and 1.5 g/kg were used for test. (a) There were significant treatment effects on tail suspension test (TST) performed 1 hour after a single administration of Yueju or fluoxetine (*F* (4, 50) = 6.267, *p* < 0.05) and (b) on forced swimming test (FST) carried out at 3 hours after administration (*F* (4, 54) = 3.551, *p* < 0.05). Mice were also tested for novelty-suppressed feeding test (NSF) at 24 hours after administration of Yueju or fluoxetine ((c) and (d)), food consumption (*F* (4, 56) = 3.530, *p* < 0.05), and latency (*F* (4, 56) = 3.317, *p* < 0.05). There was no significant treatment effect on total distance (*F* (4, 45) = 0.2248, *p* > 0.05) and time spent in central areas (*F* (4, 45) = 0.2663, *p* > 0.05) of open field test (OFT) measured for 5 minutes ((e) and (f)). Data represent means ± SEM. ^*∗*^*p* < 0.05, compared with control group (one-way ANOVA and Tukey's post hoc test). *n* = 10–13/group.

Antidepressant response was profoundly influenced by the mouse strain [19], and thus the optimal antidepressant dose of Yueju in the ICR strain mice was screened. The dose range from 1 g to 1.5 g/kg in mice was approximate to the routine OCT use of Yueju in human, which was selected for testing antidepressant response. Fluoxetine was used as a positive control with a dose of 18 mg/kg. Animals were given a single dose of Yueju or fluoxetine and the immediate and persistent anti-depressant activity was tested by using the behavior paradigms of open field test, tail suspension test, forced swim test, and novelty-suppressed feeding test. Only 1.25 g/kg of Yueju significantly reduced immobility time in the TST (*p* < 0.05) at 1 h and FST (*p* < 0.05) at 3 h after administration (Figures 1(a) and 1(b)). This dose of Yueju also increased the food consumption (*p* < 0.05, Figure 1(c)) and reduced the latency to eat (*p* < 0.05, Figure 1(d)) in NSF at 24 h. Fluoxetine effectively decreased the immobility time in the TST at 1 h (*p* < 0.05, Figure 1(a)). However, it failed to induce antidepressant response on FST at 3 h after administration or subsequent behavioral tests in NSF (*p* > 0.05, Figure 1(b)). The administration of any dose of Yueju or fluoxetine did not affect the time spent in central area or total distance traveled in the open field test (Figures 1(e) and 1(f)). Therefore, the dose of 1.25 g/kg was used in the following experiments.

### 3.2. Antidepressant Effects of Yueju Lasted Longer than Fluoxetine in the Chronic Learned Helplessness Mice

**Figure 2:**
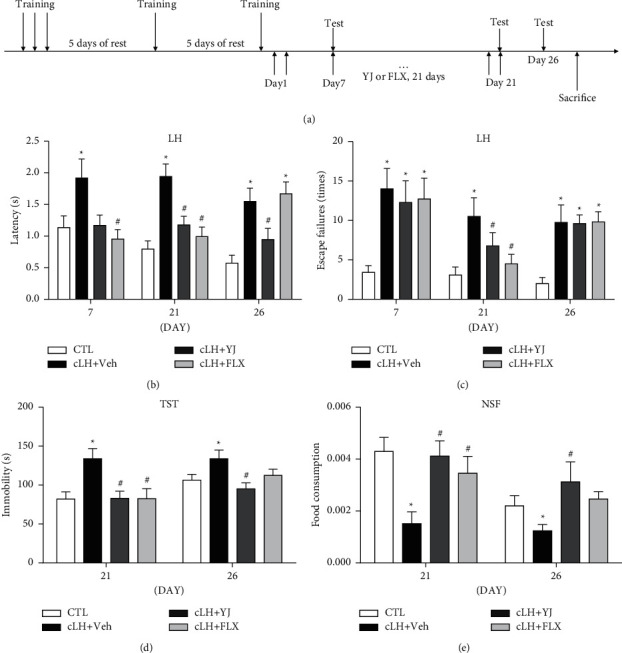
Depression-related behavioral effects after fluoxetine or Yueju treatment following chronic learned helplessness procedure. The timeline of the chronic learned helplessness (cLH) procedure is illustrated (a). Control animals (CTL) received vehicle treatment and no training, and animals exposed to cLH received administration of vehicle (Veh), Yueju (YJ), or fluoxetine (Flx) for 21 days. ((b) and (c)) Escape failures and latency to escape were measured on days 7, 21, and 26 following the 3-day plus 2 intermittent training sessions. There were significant treatment effects on latency (*F* (3, 44) = 4.924 for day 7, *F* (3, 41) = 11.08 for day 21, and *F* (3, 41) = 10.37 for day 26, *p* < 0.05) and escape failures (*F* (3, 41) = 4.669 for day 7, *F* (3, 41) = 5.990 for day 21, and *F* (3, 41) = 7.559 for day 26, *p* < 0.05). (d) Immobility time in tail suspension test (TST) (*F* (3, 42) = 4.966, *p* < 0.05 for day 21, *F* (3, 40) = 3.501, *p* > 0.05 for day 26) and food consumption (*F* (3, 39) = 5.240, *p* < 0.05 for day 21, *F* (3, 30) = 6.030, *p* < 0.05 for day 26) in the novelty-suppressed feeding test (NSF) were measured for 10 minutes (e). Data are expressed as mean ± S.E.M. ^*∗*^*p* < 0.05, compared to CTL; ^#^*p* < 0.05, compared to Veh (one-way ANOVA and Tukey's post hoc test). *n* = 8–12/group.

Animals received administration of Yueju or fluoxetine following the chronic learned helplessness (cLH) paradigm. They were tested for LH response on day 7 (short-term drug treatment), day 21 (long-term treatment time), and day 26 (5 days after termination of 3-week drug treatment), respectively, in the independent cohorts. OFT, TST, and NSF behaviors were additionally examined in animals receiving long-term treatment of drugs (Figure 2(a)). In the cLH paradigm, animals receiving vehicle treatment displayed learned helpless behavior on day 7, day 21, and day 26. One week after treatment, the latency to escape (*p* < 0.05 versus Veh), but not the escape failures of LH, was improved by fluoxetine, whereas neither of them was improved by Yueju. Following 3 weeks of administration, both Yueju and fluoxetine improved latency to escape (both *p* < 0.05 versus Veh) and escape failures (both *p* < 0.05 versus Veh) (Figures 2(b) and 2(c)).

On day 26, or 5 days after discontinuation of drugs, animals previously treated with fluoxetine failed to reverse any measurement in learned helplessness (Figures 2(b) and 2(e), both *p* > 0.05 versus Veh). In contrast, mice previously treated with Yueju still showed efficacy in the measurement of latency to escape (*p* < 0.05versus Veh). The cLH exposed animals also displayed other depressive-like responses, indicated by increased immobility time in TST and reduced food consumption in NSF (all *p* < 0.05 versus CTL). Yueju significantly reduced the immobility time in TST (*p* < 0.05) and increased food consumption (*p* < 0.05) in NSF. In contrast, chronic fluoxetine neither attenuated the immobility time in TST (*p* > 0.05) nor improved food consumption in NSF (*p* > 0.05, Figures 2(d) and 2(e)). No difference in the total distance traveled or central time in OFT (Supplementary Figure S3, *p* > 0.05 versus Veh) was found at this time. In summary, 3-week administration of conventional Yueju elicited a long-lasting antidepressant effect, whereas 3 weeks of fluoxetine treatment was effective but the effect was not persistent, indicating a relapse with this treatment.

### 3.3. Yueju Treatment Upregulated PKA-CaMKII Signaling and Downregulated NR1 Expression in Learned Helplessness Mice

**Figure 3:**
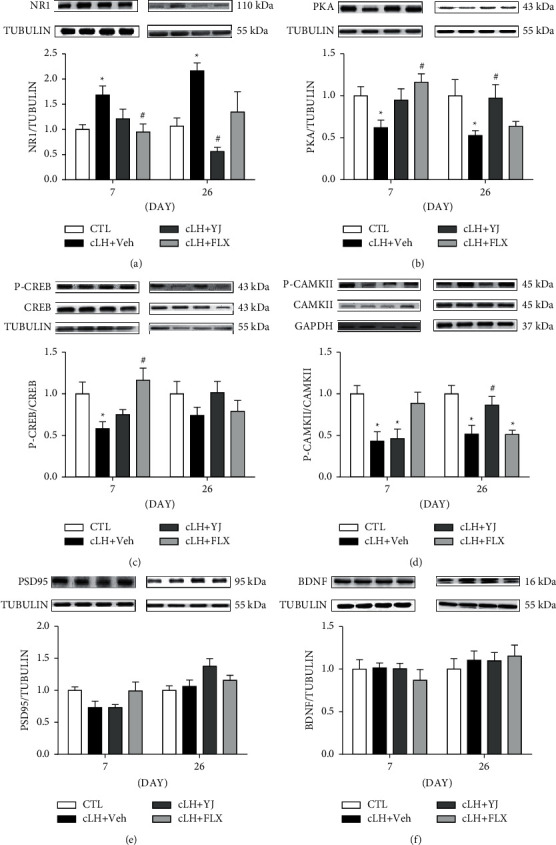
Western blots of neural plasticity related signaling in the hippocampus. Control animals (CTL) received vehicle treatment and no training, and animals exposed to chronic learned helplessness (cLH) for 21 days received administration of vehicle (Veh), Yueju pills (YJ), or fluoxetine (Flx). (a) Protein expression levels of NR1 (*F* (3, 21) = 3.985 for day 7; *F* (3, 20) = 8.190 for day 26, *p* < 0.05). (b) PKA (*F* (3, 22) = 3.526 for day 7; *F* (3, 20) = 3.186 for day 26, *p* < 0.05), (c) P-CREB/CREB (*F*(3, 24) = 5.60 for day 7, *p* < 0.05; *F* (3, 19) = 1.182 for day 26, *p* < 0.05), (d) P-CaMKII/CaMKII (*F* (3, 21) = 6.565 for day 7; *F* (3, 18) = 8.607 for day 26, *p* < 0.05), (e) PSD95F (3, 20) = 2.770 for day 7; *F* (3, 20) = 3.080 for day 26, *p* > 0.05), and (f) BDNF (*F* (3, 20) = 0.5380 for day 7; *F* (3, 20) = 0.3171 for day 26, *p* > 0.05) in the hippocampus. Data are expressed as mean ± S.E.M. ^*∗*^*p* < 0.05, compared to CTL; ^#^*p* < 0.05, compared to Veh (one-way ANOVA and Tukey's post hoc test). *n* = 6–8/group.

The molecular signaling responsible for hippocampal neural plasticity, including PKA-CREB, CaMKII, and a NMDA receptor subunit NR1, was examined at 1 week or 5 days following discontinuation of 3 weeks of drug treatment in mice exposed to cLH protocol. By 1 week after cLH and drug administration, NMDA subunit NR1 expression was upregulated, while PKA, pCREB/CREB, and p-CaMKII/CaMKII signaling were downregulated in the hippocampus (all *p* < 0.05). Fluoxetine normalized the NR1 and PKA-CREB levels, whereas Yueju failed to show improvement on these signaling expressions. By 26 days after cLH when drugs were discontinued for 5 days, there were still abnormal expressions of NR1, PKA, and CaMKII (all *p* < 0.05). Animals previously treated with Yueju still showed improvement in expression levels of NR1, PKA, and p-CaMKII/CaMKII (all *p* < 0.05). In contrast, none of the expressions of these proteins were improved at 5 days after discontinuation of fluoxetine. The expression of p-CREB/CERB was not significantly different by the treatment at this time (Figures 3(a)–3(d), *p* > 0.05). There was no difference in the expressions of PSD95 and BDNF across different groups at either of the time points (Figures 3(e)–3(f), *p* > 0.05).

### 3.4. Blockade of CaMKII Signaling Blunted Antidepressant Effects of Yueju

**Figure 4:**
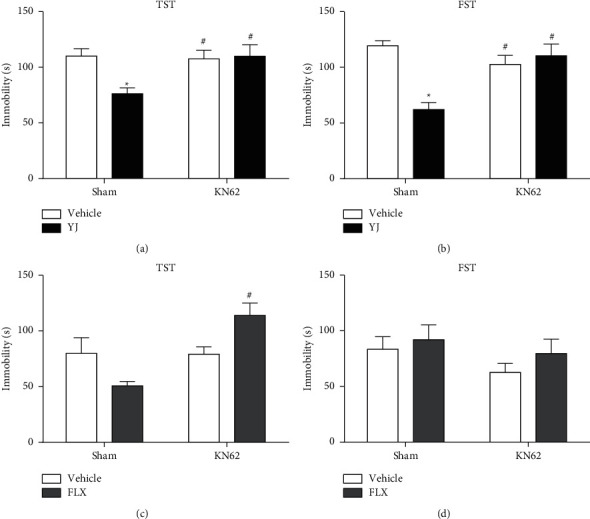
Intracerebroventricular microinjection of KN-62 blocked Yueju induced antidepressant effect. Mice received intracerebroventricular injection of vehicle or KN-62 (5 *μ*mol/site) 30 min prior to Yueju or fluoxetine. Four groups of mice were used to investigate the involvement of CaMKII signaling in the antidepressant-like effect of Yueju or fluoxetine: (1) vehicle plus Sham, (2) vehicle plus KN-62, (3) Yueju or fluoxetine plus Sham, and (4) Yueju or fluoxetine plus KN-62. (a) Tail suspension test (TST) was performed 1 h after the administration of Yueju. (b) Forced swimming test (FST) was performed 3 h after the administration of Yueju. (c) TST was performed 1 h after the administration of fluoxetine. (d) FST was performed 3 h after the administration of fluoxetine. ^*∗*^*p* < 0.05 compared with vehicle + Sham group; ^*#*^*p* < 0.05 compared with Yueju + Sham group (two-way ANOVA and Tukey's post hoc test).

As normalization of CaMKII signaling was critically associated with the sustained antidepressant effect of Yueju, we assessed the role of its activation in the antidepressant response of Yueju as well as fluoxetine. Mice first received intracerebroventricular injection of an antagonist of CaMKII, KN-62 (1 *μ*g/5 *μ*l), or vehicle and 30 min later were given intragastric administration of Yueju/fluoxetine or saline. There was a significant effect of KN-62 and Yueju interaction (Figure 4(a), *F* (1, 43) = 5.818, *p* < 0.05 for TST; *F* (1, 40) = 17.65, *p* < 0.05 for FST). Post hoc analyses showed that the administration of KN-62 alone did not affect the immobility in the TST or FST in non-Yueju mice (*p* > 0.05). However, it blunted the reduction of immobility time in TST or FST by Yueju (both *p* < 0.05). In contrast, KN-62 could not block the antidepressant effect of fluoxetine: a two-way ANOVA revealed the significant interaction between fluoxetine and KN-62 treatment (Figure 4(b), *F* (1, 42) = 10.26, *p* < 0.05) for immobility time in TST, but post hoc analyses showed no difference between any two groups. No main or interaction effect was found between fluoxetine and KN-62 treatment in FST (*F* (1, 41) = 0.1262, *p* > 0.05). The four groups have no main effect in open field test for total distance (*p* > 0.05) and time spent in central area (*p* > 0.05) (Figures 4(c)–4(d), 4G-H). All of the treatments did not change the OFT behavior (Supplementary Figure S4).

## 4. Discussion

The present study aimed to test the ability to maintain antidepressant activity following a chronic treatment and the associated signaling in a long-term learned helplessness depression model. We found that both Yueju and fluoxetine produced antidepressant response following 3 weeks' treatment. Five days following termination of drug administration, only Yueju continued to show antidepressant effects. Yueju, but not fluoxetine, reversed the deficient expressions of PKA, NR1, and CaMKII signaling in the hippocampus of cLH mice. The antidepressant effect of Yueju, but not fluoxetine, was blocked after the i.c.v. administration of CaMKII antagonist (KN-62). Together, antidepressant effects of Yueju lasted longer than fluoxetine, associated with the sustained improvement of CaMKII signaling.

Major depression is characteristic of the long-term illness condition. Here, in the chronic learned helplessness model, animals showed the depression-like behaviors at least for 26 days, as reported previously in other strains of mice [19]. This long-term disease status allows us to evaluate the maintenance of antidepressant activity of the drugs in the depression-like condition. Three-week chronic administration of Yueju or fluoxetine produced comparable antidepressant effects but the ability to maintain the antidepressant activity was different. Repeated administration of Yueju lasted for at least 5 days after discontinuation, not only in the tests of learned helplessness but also in the other depression-related paradigms including behavior despair and NSF. This data may reflect the clinic observation that SSRI was prone to relapse after discontinuation [34, 35]. The vulnerability to relapse/recurrence remains a major challenge for current SSRI treatment of depression [36, 37]. The maintenance of the antidepressant activity following termination of Yueju suggests that this drug may have better treatment outcome in reducing the risk of relapse/recurrence. This certainly warrants further clinical investigation.

Disruption in cyclic cAMP-PKA signaling has been implicated in stress maladaptation, anxiety, and depression [23]. Chronic treatment with fluoxetine enhances cAMP levels, subsequently activates PKA, and upregulates CREB signaling in the hippocampus, cortex, and hypothalamus of the chronically stressed rats [27]. The previous studies showed that Yueju treatment induced an upregulation of PKA-CREB signaling expression in the KM mice [19]. The level of PKA protein in the hippocampus of mice increased on day 7 by fluoxetine and at 5 days following termination of Yueju on day 26, in agreement with their respective restoration of behaviors. Yueju also showed the tendency to upregulate CREB signaling, although the CREB signaling was restored, consistent with the restored expression of the downstream BDNF and PSD95 at this time. The strain difference may account for the difference in the expressions of PSD95 and BDNF between this study and the previous study. In this study, the ICR strain mice were used, whereas Kunming strain mice were used in the previous study [19]. It has been demonstrated from a previous study that there are strain-dependent differences in the expressions of PKA, CREB, and BNDF, in which ICR mice were relatively insensitive to Yueju pills or ketamine, as compared with Kunming mice [22]. Additionally, this strain difference may also account for the difference in the optimal doses for the antidepressant activity of Yueju [19]. Recent studies demonstrate that ketamine, an NMDAR antagonist, plays an important role in synaptic plasticity [38, 39]. The antidepressant effect of repeated administration of Yueju is closely related to NMDA receptors in KM mice [19]. This study provided a further evidence that upregulation of NR1 was associated with relapse of depression following fluoxetine treatment, whereas prevention of relapse with Yueju treatment was associated with downregulation of NR1. Therefore, this study supports that deficiency/restoration of PKA signaling and NR1 expression was associated with the relapse/sustainability of antidepressant effects, respectively.

This study highlighted the contribution of CaMKII signaling to the antidepressant effect of Yueju and its prevention of relapse. Ca^2+^/calmodulin-dependent protein kinase II (CaMKII) is a multifunctional serine/threonine kinase which is involved in synaptic plasticity [40]. The activation of CAMKII was shown to participate in CREB signaling as well as other signalings, and CaMKII signaling was abnormal in both central and peripheral tissues of depressive patients or animals submitted to models of depression [34, 41]. Acute imipramine and electroconvulsive treatment increased CAMKII activity in the hippocampus of mice [42]. The present results showed that the expression of CaMKII in the hippocampus of mice was significantly reduced after cLH; administration of fluoxetine resulted in a trend of improvement on day 7 and was reversed at 5 days following discontinuation of Yueju, which was consistent with their behavioral responses, respectively. This suggests that the difference in the regulation of CaMKII expression between the two drugs may account for the differentiated behavioral response overtime, and sustained antidepressant effect of Yueju was associated with sustained restoration of CaMKII signaling. Furthermore, after pretreatment with CaMKII blocker KN-62 in the mouse brain, Yueju did not produce antidepressant effect anymore, indicating the dependence of antidepressant effect of low dose of Yueju on CaMKII signaling activation. After 7 days of daily treatment of fluoxetine, the expression of CaMKII was normalized, and there was a sign of antidepressant effect of fluoxetine, which suggests that the normalization of CaMKII level was associated with the antidepressant response. However, when a single treatment of fluoxetine was used, it failed to induce antidepressant effect; therefore, it was not possible to assess whether the CaMKII signaling was responsible for fluoxetine's effects. Our results support the fact that CaMKII may play an important role in the pathophysiology and treatment of depression.

It was worth noting that when we screened the optimal dose of Yueju in ICR mice here, we found that 1.25 g/kg of Yueju showed both immediate and a short-term antidepressant activities, whereas 1.5 g/kg only showed a late-onset antidepressant activity and the low dose of 1 g/kg was ineffective. These results indicated a U-shape dose-effect relationship, which was not uncommon for a herbal formula and has been demonstrated in our previous studies [43]. One interpretation is that the compounds responsible for the antidepressant effects may be dose-sensitive and only have a narrow optimal dose range. Alternatively but not mutually exclusively, with the dose of 1.5 g/kg of Yueju, the concentration of some counteractive compounds was sufficient to attenuate the antidepressant actions of the effective compounds. More studies should address the underlying mechanism.

In summary, this study showed that the chronic administration of conventional low dose of Yueju elicited sustained antidepressant effects in a chronic depression model, associated with a long-term restoration of PKA/CaMKII and NMDA signaling in the hippocampus. The activation of CaMKII signaling is required for the antidepressant response of Yueju. The maintenance of antidepressant activity and molecular signaling by Yueju may indicate its capacity to better counteract relapse. Further study of the mechanism of relapse and its prevention is of vital importance to improve the current treatment of depression.

## Data Availability

All datasets used in this study are included in the manuscript and the supplementary files.

## Supplementary Materials

Supplementary Materials Supplementary Figure S1: the HPLC fingerprint of standard substances (A); the HPLC fingerprint of Yueju pill extracts (B); chlorogenic acid (1), geniposide (2), ferulic acid (3), senkyunolide I (4), senkyunolide H (5), coniferyl ferulate (6), senkyunolide A (7), n-butylphthalide (8), ligustilide (9), 3-butylidenephthalide (10), and atractylodin (11). Supplementary Figure S2: the structure of major compounds in Yueju pills extracts. Supplementary Figure S3: total distance (*F* (3, 28) = 0.2375, *p* > 0.05 for day 21, *F* (3, 28) = 0.01572, *p* > 0.05 for day 26) and time spent in central areas (*F* (3, 27) = 0.7848, *p* > 0.05 for day 21, *F* (3, 28) = 1.465, *p* > 0.05 or day 26) of open field test (OFT) were measured for 5 min. Supplementary Figure S4: time spent in central areas (*F* (1, 44) = 0.2590, *p* > 0.05 for A, *F* (1, 44) = 0.1159, *p* > 0.05 for C) and total distance (*F* (1, 44) = 1.794, *p* > 0.05 for B, *F* (1, 41) = 0.1950, *p* > 0.05 for D) of open field test (OFT) were measured for 5 minutes.
