# Supplementary material for: Repeated Yueju, But Not Fluoxetine, Induced Sustained Antidepressant Activity in a Mouse Model of Chronic Learned Helplessness: Involvement of CaMKII Signaling in the Hippocampus
Source: Evid Based Complement Alternat Med. 2022 Feb 24;2022:1442578. doi: 10.1155/2022/1442578 (PMC8894000; doi:10.1155/2022/1442578)
Supplement: Supplementary Materials — Supplementary Figure S1: the HPLC fingerprint of standard substances (A); the HPLC fingerprint of Yueju pill extracts (B); chlorogenic acid (1), geniposide (2), ferulic acid (3), senkyunolide I (4), senkyunolide H (5), coniferyl ferulate (6), senkyunolide A (7), n-butylphthalide (8), ligustilide (9), 3-butylidenephthalide (10), and atractylodin (11). Supplementary Figure S2: the structure of major compounds in Yueju pills extracts. Supplementary Figure S3: total distance (F (3, 28) = 0.2375, p > 0.05 for day 21, F (3, 28) = 0.01572, p > 0.05 for day 26) and time spent in central areas (F (3, 27) = 0.7848, p > 0.05 for day 21, F (3, 28) = 1.465, p > 0.05 or day 26) of open field test (OFT) were measured for 5 min. Supplementary Figure S4: time spent in central areas (F (1, 44) = 0.2590, p > 0.05 for A, F (1, 44) = 0.1159, p > 0.05 for C) and total distance (F (1, 44) = 1.794, p > 0.05 for B, F (1, 41) = 0.1950, p > 0.05 for D) of open field test (OFT) were measured for 5 minutes. [file 1442578.f1.docx]

**The HPLC fingerprint of Yueju pill extracts**

The sample was prepared as following procedure: 2 g of Yueju pills were dissolved in 10 mL of 70% methanol and extracted for 30 min. The sample was analyzed by using high performance liquid chromatography (HPLC). The HPLC analysis was performed on a Waters 2695 system (Waters Corporation, Milford, MA, USA), consisting of a binary solvent delivery manager, an auto-sampler and a PDA detector. Chromatographic separations were performed on Alltima C_18_ column (5 μm, 250×4.6 mm). Flow rate and column temperature were set at 1 mL·min-1 and 30 °C, respectively. A mobile phase system consisting of 0.1% formic acid in H_2_O (A)-Acetonitrile (B) was applied with the following gradient program: 0-8 min, 90%A; 8-15 min, 90-80% A; 15-25 min, 80% A; 25-30 min, 80%-70% A; 30-38 min, 70% A; 38-43 min, 70%-50% A; 43-48 min, 50% A; 48-56 min, 50%-30% A; 56-59 min, 30% A; 59-65 min, 30%-0% A; 65-75 min, 0% A; 75-80 min, 0%-90% A.


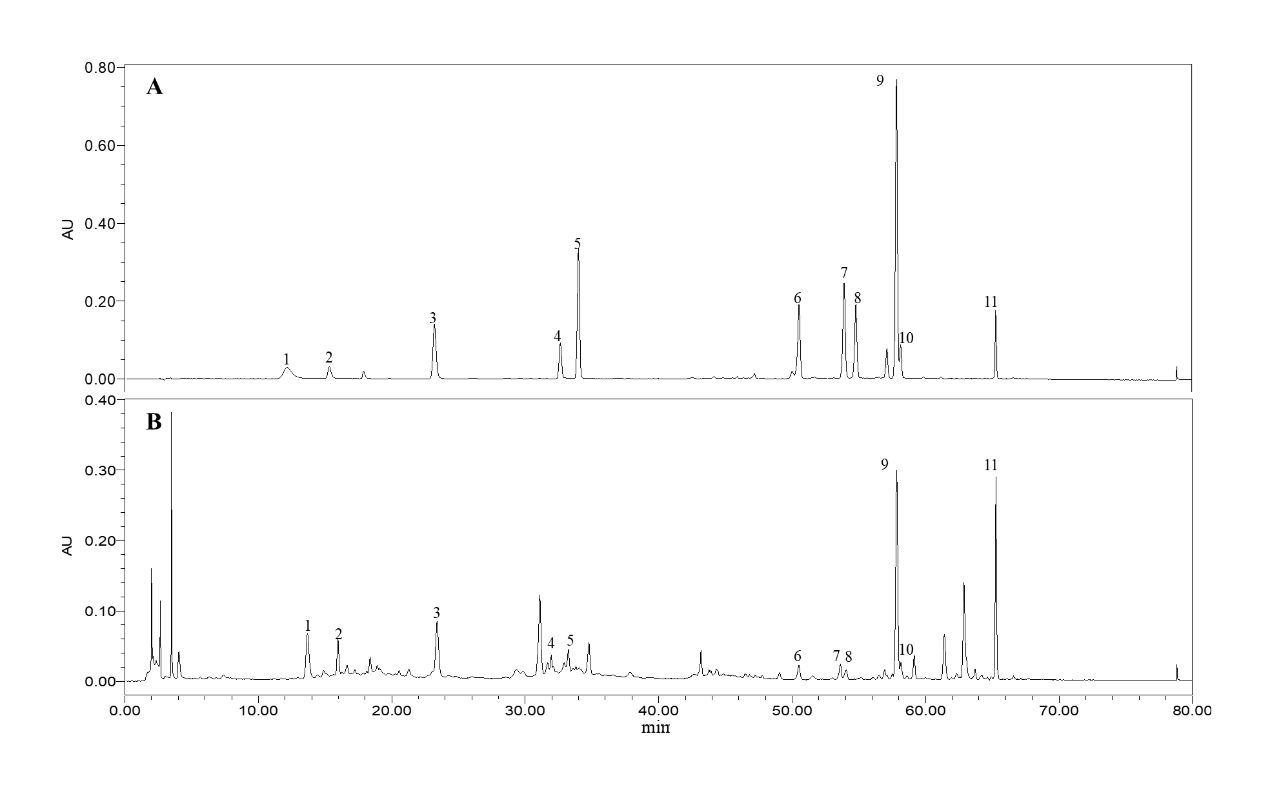


**Supplementary Figure S1: The HPLC fingerprint of standard substances (A); The HPLC fingerprint of Yueju pill extracts (B);** chlorogenic acid (1), geniposide (2), ferulic acid (3), senkyunolide [I](https://www.chemsrc.com/en/cas/63038-10-8_724592.html) (4), senkyunolide H (5), coniferyl ferulate (6), senkyunolide [A](https://www.chemsrc.com/en/cas/63038-10-8_724592.html) (7), n-butylphthalide (8), ligustilide (9), 3-butylidenephthalide (10), atractylodin (11).





**Supplementary Figure S2**: The structure of major compounds in Yueju pills extracts.


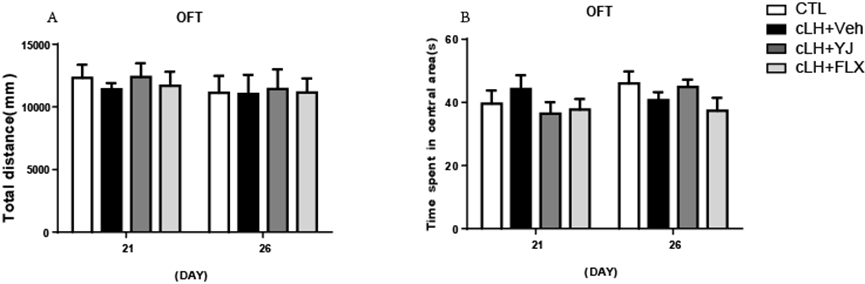


**Supplementary Figure S3**: Total distance (F (3, 28) = 0.2375, *p* >0.05 for Day 21, F (3, 28) = 0.01572, *p* >0.05 for Day 26) and time spent in central areas (F (3, 27) = 0.7848, p> 0.05 for Day 21, F (3, 28) = 1.465, *p* >0.05 or Day 26) of open field test (OFT) were measured for 5 min.


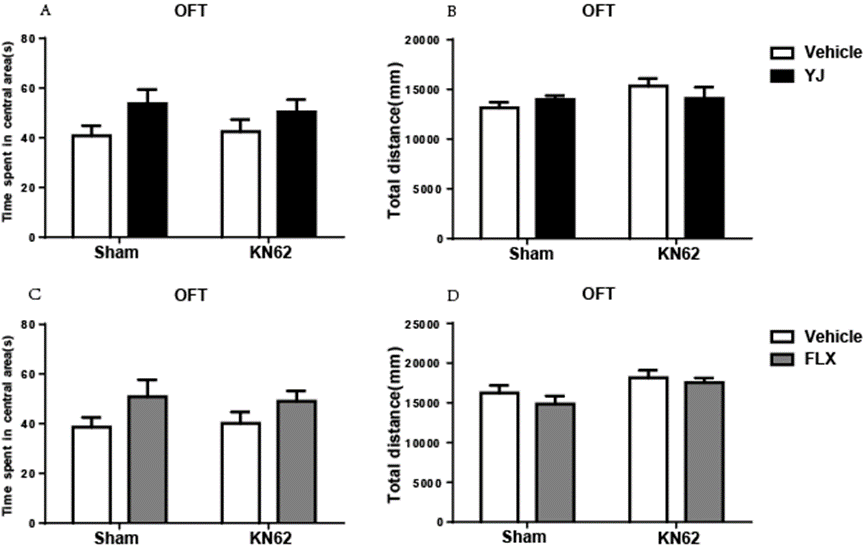


**Supplementary Figure S4**: Time spent in central areas(F (1, 44) = 0.2590, *p* > 0.05 for A, F (1, 44) = 0.1159, *p* > 0.05 for C) and total distance(F (1, 44) = 1.794, *p* >0.05 for B, F (1, 41) = 0.1950, *p* > 0.05 for D) of open field test(OFT) were measured for 5 minutes
